# Clinical and surgical characteristics of infected diabetic foot ulcers in a tertiary hospital of Mexico

**DOI:** 10.1080/2000625X.2017.1367210

**Published:** 2017-09-06

**Authors:** Estrella Cervantes-García, Paz María Salazar-Schettino

**Affiliations:** ^a^ Facultad de Medicina, Universidad Nacional Autónoma de México, Ciudad de México, Mexico

**Keywords:** Diabetes, diabetic foot, surgical washing, ulcers

## Abstract

**Background**: The objective of this study was to determine the clinical and surgical characteristics of diabetic foot ulcers in a tertiary level hospital in Mexico.

**Methods**: We performed a longitudinal, descriptive study from July, 2012 to August, 2015 on a sample composed of 100 patients with type 2 diabetes mellitus and infected diabetic foot ulcers. We analyzed socio-demographic variables, comorbidities, characteristics of ulcers, and the applied treatment.

**Results**: We found that the most affected areas were the forefoot (48%) and the plantar region (55%) of the foot. Also, most of the patients arrived with advanced stages of diabetic foot ulcers, since 93% of the lesions were of grades III–V according to the Wagner classification. Moreover, lesions usually present with advanced states of infection, since 60% of the lesions were of grades 3–4 in the PEDIS scale. In addition, the great majority of the patients are prone to complications because we found that 43% of the patients suffered from hypertension, 47% of the patients had chronic kidney disease, and 45% reported smoking. In fact, 45% of the patients eventually suffered an amputation. We also found that the situation is more difficult because the great majority of the patients (96%) have a low level of education and very low income and they do not have any health insurance. Nevertheless, we also found that an efficient treatment can help in avoiding amputations, since 53% of grade IV and 25% of grade V lesions according to the Wagner system did not suffer an amputation.

**Conclusions**: Therefore, an effective antibiotic treatment and an education of the patient on the adequate care of their lesions are essential in increasing the welfare of patients, especially when they have a low level of education.

## Introduction

Diabetic foot is one of the most frequent and devastating complications of patients with type 2 diabetes mellitus (T2DM). It constitutes a serious problem for public health systems due to high treatment costs and it is one of the principal causes of morbidity, mortality, and disability [1–3]. In addition, it also has a negative impact on quality of life and constitutes a heavy socio-economic burden on the patient and the community, since it entails the risk of losing the extremity and, thus, usually requires a prolonged hospital stay [4–7].

The foot ulcers commonly developed by diabetic patients are located on the plantar surface of the foot [6,7]. These lesions can become chronic and can lead to an amputation because of the delayed healing suffered by diabetic patients. The factors associated with a diabetic foot ulcer depend on its etiology, such as neuropathy, ischemia, and infection [8–10]. Patients with neuropathy and insufficient peripheral artery flow have a greater risk of developing ulcers than those that only have peripheral diseases [6,7]. On the other hand, patients with neuropathy have more possibilities of healing an ulcer than those with an artery disease [10–12].

Proper care of diabetic foot ulcers requires a clear and descriptive classification of the lesions. This system must be used to guide physicians towards an adequate treatment of each wound, aside from a certain ability to predict prognosis in each case. Multiple classification systems for diabetic foot wounds have been put forward [13–16]. However, none are considered as the definite one. One that is frequently used is the Wagner system, which classifies foot ulcers according to the depth of the lesion and may support the prediction of amputation associated to other agents [17]. Another classification frequently used for the diagnosis of diabetic foot infection is the PEDIS system developed by The International Working Group on the Diabetic Foot (IWGDF) [18,19]. PEDIS itself stands for perfusion, extent (size), depth (tissue loss), infection, and sensation (neuropathy).

The objective of this study was to determine the clinical and surgical characteristics of infected diabetic foot ulcers in patients with T2DM in a tertiary level hospital in Mexico.

## Materials and methods

A longitudinal, descriptive study was performed from July 2012 to August 2015 on patients admitted to the Emergency Room of the Hospital General de México. The sample consisted of 100 patients diagnosed with T2DM and infected diabetic foot ulcers. A wound was defined using the *International Consensus on the Diabetic Foot* as a full-thickness wound below the ankle in a diabetic patient, irrespective of the duration, tissue necrosis, and gangrene. It is important to note that all the patients had already received empiric antibiotic treatment before our study.

### Data collection

First, we informed the patients about the study, its objectives, and relevance. Afterwards, we asked for their consent. Then, those patients that did accept to participate in the study were interviewed and answered a questionnaire in face to face interviews. The questionnaire included the following socio-demographic and economic data: gender, age, educational level, if they had a job or not, and income. It also included the following clinical data: medical history, smoking status, alcoholism, access to health care centers, history of foot problems, current foot or leg problems, years with diabetes, treatment of diabetes, duration of the ulcers, albumin, white blood cell count, and kidney disease classified in K/DOQI (Kidney Disease Outcomes Quality Initiative).

In addition, we performed a physical examination to document the obesity and we recorded ulcer location, number of affected zones, infection, edema, depth and area of the ulcer, and the type (minor or major) of amputation if the patients eventually suffered one.

The ulcers were classified using the Wagner system which uses the following grades:

- Grade 1: ulcerated skin and subcutaneous tissue;
- Grade 2: deep lesions that could penetrate to the tendon or joint capsule but not the bone (there is no abscess or osteomyelitis);
- Grade 3: deep tissues are involved with abscess or osteomyelitis;
- Grade 4: localized gangrene;
- Grade 5: generalized gangrene.

Also, the diagnosis of diabetic foot infection was made using the PEDIS system developed by IWGDF. This scale classifies the lesions as follows:

- Grade 2: mild infection of the skin/subcutaneous tissue;
- Grade 3: moderate or mild with erythema >2 cm or infection of structures deeper than the subcutaneous tissue;
- Grade 4: severe infection with systemic signs of inflammation.

On the other hand, minor amputations were defined as follows:

- partial toe amputation,
- complete toe disarticulation at the metatarsophalangeal joint,
- ray (toe and metatarsal amputation), or
- proximal foot amputation (transmetatarsal).

Major amputations were those that occur at the proximal to the tarsometatarsal joint:

- Chopart,
- Boyd,
- Syme,
- below the knee,
- above the knee.

We also measured the glycated hemoglobin (HbA1c) and diagnosed hypertension. Hypertension was diagnosed if one of the following conditions was present: systolic blood pressure >140 mmHg, diastolic blood pressure >90 mmHg, or if the patient used antihypertensive medication.

All patients received *surgical washing* every day, three times a week, or once a week depending on the severity of the lesions. By *surgical washing* we mean the following treatment. We first washed the lesions vigorously with a sterile sponge or gauze soaked with physiologic saline or serum. Then, the lesions were vigorously washed with surgical soap (chlorhexidine or glycerin soap). Afterwards, the lesions were vigorously washed again with a sterile sponge or gauze soaked with physiologic saline or serum. This was done to remove all contaminants and the soap from the lesion. Once this was done, we practiced (when necessary) a debridement with a scalpel to remove all the border of tissue, hyperkeratosis, callus, and dead or necrotic tissue. After the debridement, we again washed the lesions vigorously with a sterile sponge or gauze soaked with physiologic saline or serum, and we then dried the foot with a sterile gauze. Then, samples were obtained from the wound depth with a sterile swab and the wound was then covered with another sterile gauze or dressing. The samples were cultured and we performed an antibiogram to determine which medication was suitable for treatment purposes.

The treatment administered to each patient was also recorded. All the patients were given topical antimicrobial agents and oral or parenteral antibiotics. The antibiotics used were the following: metronidazole, clindamycin, amoxicillin/clavulanate, trimethoprim-sulfamethoxazole, ciprofloxacin, imipenem, quinolones, and linezolid. In addition, four patients received vancomycin.

For statistical analysis we used SPSS 24.

The study was approved by the Ethics Committees of the Universidad Nacional Autónoma de México (UNAM) and the Hospital General de México.

## Results

The sample consisted of 100 patients admitted to the Emergency Room of the Hospital General de México. The patients were diagnosed with T2DM and infected diabetic foot ulcers.

Table 1 shows the socio-demographic characteristics of the patients. First, we note that only 15% of the patients were overweight (body mass index greater than or equal to 25) and that the average duration of T2DM for the sample was 10 years. Observe that 60% of the patients where male and that the average age of the patients was 52 years for men and 50 for female. We found that all the patients were between 20 and 79 years old. In addition, note that most of the patients had a low level of schooling since 76% of them were either illiterate or only had (a complete or incomplete) elementary education (grades 1–6) and only 4% of them had a university degree.

**Table 1.** Demographic data (*n* = 100 patients).

Body mass index	21.4 ± 5.2	
Duration of DM2	10 ± 5.2 (years)	
Gender	Percentage	Average age (years)
Female	40%	50 ± 10.4
Male	60%	52 ± 10.2
Elementary education	Males	Females
(grades 1–6)	(% of 100 patients)	(% of 100 patients)
Incomplete	14%	3%
Complete	12%	10%
Middle education		
(grades 7–9)		
Complete	8%	3%
Incomplete	2%	1%
High school education		
(grades 10–12)		
Incomplete	5%	1%
University degree	1%	3%
Illiterate	18%	19%
Total	60%	40%

Table 2 shows risk factors for the development of diabetic foot ulcers. In particular, it presents the average leukocytosis and serum albumin of the sample at the moment of the first evaluation. Notice that the average leukocytosis was high (>9) and that the average serum albumin was low (<2). Also, hypertension was present in 43% of the patients and the HbAc1 was high (>7) in 85% of the patients. Moreover, observe that the great majority (75%) of the patients took medication with some regularity to control the glycemia. We note that many of the patients could not follow the treatment adequately for economic reasons. Table 2 also shows that 47% of the patients presented with chronic kidney disease in K/DOQI and that K/DOQI-III was the most common. Finally, notice that smoking was present in 45% of the patients and that alcoholism was present in 32%.

**Table 2.** Risk factors (*n* = 100 patients).

Leukocytosis	12.780 ± 3.386
Serum albumin	<2
Hypertension	% of patients
	43%
HbAc1	85%
Treatment for diabetes	
Oral	55%
Parenteral/insulin	20%
No treatment	25%
Chronic kidney disease	
K/DOQI II	10%
K/DOQI III	25%
K/DOQI IV	12%
Smoking	45%
Alcoholism	32%

Table 3 describes the characteristics of the diabetic foot ulcers. It was observed that the lower right extremity was affected in 67% of cases, while the lower left extremity presented with a lesion in 33% cases. We note that there were no cases in which both feet presented with an ulcer. Moreover, the area of the ulcer varied from 1.5 to 8 cm^2^ (the area was calculated by multiplying the two maximum dimensions at right angles).

**Table 3.** Clinical characteristics of the ulcers (*n* = 100 patients).

Variable	*n* = 100 Patients
Damaged foot	%
Right	67
Left	33
Size of ulcer	1.5–8 cm^2^
Triggering event	%
Traumatic	16
Accidental injury	14
Blisters	20
Trauma due to inadequate shoes	25
Callus	10
Ingrown toenails	10
Pruritus	5
Other characteristics	%
Anhidrosis (absence of sweat)	40
Erythema	24
Edema	15
Decrease sensitivity	17
Hypothermia	4
Cultures of the wound infection	%
*S. aureus*	42
*E. coli*	36
Others bacteria	47

Table 3 also reports the triggering events for the development of the diabetic foot ulcers. We found that the two main causes were the use of inappropriate shoes (25%) and the formation of blisters (20%). Table 3 also presents other characteristics that were found in the foot lesions. Of these, anhidrosis (deficiency or absence of sweat) (40%) and erythema (24%) were the most common. It is interesting to note that a total of 4 patients presented with hypothermia in the moment of consultation.

Table 3 also reports the results of the cultures of the wound infection. We found methicillin-resistant *Staphylococcus aureus* (MRSA) in 42% of the samples, followed by *Escherichia coli* in 36% and, in lower percentages, other bacteria. It is important to note that most cultures were polymicrobial.

Table 4 compares the characteristics of the ulcers according to the treatment received by the patients: only surgical washing with antimicrobial treatment or eventual surgical treatment (minor or major amputation performed). First, observe that it presents the average leukocytosis after the patients received an efficient treatment including surgical washing and antibiotics using an antibiogram. The average of leukocytosis was high in patients that suffered an amputation (>12). Furthermore, notice that 45% of the patients had an amputation and these had an average duration of T2DM of 13 years, more than twice the average duration of T2DM in those patients that only had surgical washing. Also, the table shows that the patients that had an amputation had an average age of 58.7 years; while those that only had surgical washing had an average age of 50 years. Moreover, observe that men were more affected by amputations, since 58.3% of men had a minor or major amputation, while only 25% of women had an amputation. According to the results obtained by the Chi-square test of Homogeneity, we found that these differences in the type of treatment (surgical washing or amputation) depending on the gender were statistically significant, χ^2^_(1,100)_ = 10.774, *p* = 0.001.

**Table 4.** Comparison of treatments (*n* = 100 patients).

Variables	Amputation	Surgical Washing	Total patients
Leukocytosis	12.712 ± 2.98	9.915 ± 3.45	–
Duration of DM2	13 ± 8.6 (years)	5.5 ± 5.47 (years)	–
Age	58.7 ± 14.7(years)	50 ± 14.9 (years)	–
Gender	No. of patients	No. of patients	
Females	10	30	40
Males	35	25	60
Hypertension	24	19	43
Smoking	25	20	45
Chronic kidney disease			
K/DOQI II		10	10
K/DOQI III	25		25
K/DOQI IV	12		12
Affected region			
Forefoot	22	26	48
Midfoot	7	6	13
Hindfoot	10	19	29
More than one area affected	6	4	10
Location of lesion			
Plantar	25	30	55
Heel	5	15	20
Dorsal	15	10	25
Wagner			
Grade II		7	7
Grade III	14	25	39
Grade IV	16	18	34
Grade V	15	5	20
PEDIS			
2	5	35	40
3	25	15	40
4	15	5	20
Amputation			
Major	15	0	15
Minor	30	0	30

Table 4 also shows that 24 of the patients that had hypertension (a total of 43) suffered an amputation, while 19 of the patients that had hypertension only received surgical washing. In addition, note that the population of smokers (a total of 45) is almost equally divided among those that suffered an amputation (25 patients) and those that only received surgical washing (20 patients).

Table 4 indicates that all the patients with kidney problems and K/DOQI-III and -IV suffered an amputation (37% of the 100 patients), while those with K/DOQI-II only had surgical washing (10%).

Table 4 also presents the localization of the foot lesions according to the type of treatment received. Observe that the most affected area was the forefoot, since it was affected in 48% of the 100 patients, 49% of the patients that suffered an amputation, and 47% of the patients that only received surgical washing. Also, notice that the ulcers were found most frequently in the plantar region of the foot, since it was affected in 55% of the 100 patients, 56% of the patients that suffered an amputation, and 55% of the patients that only received surgical washing.

Table 4 shows the classification of foot lesions according to the Wagner system. Taking both groups into consideration, it was found that grades III (39%) and IV (34%) were the most common. In particular, observe that grade IV was the most frequent one in patients that suffered an amputation (36% of patients that suffered an amputation), followed closely by grades V and III (33% and 31%, respectively, of the patients that suffered an amputation). Moreover, observe that grades III and IV were the most common types of lesions among the patients that only received surgical washing (45% and 33%, respectively, of the patients that only received surgical washing).

Table 4 also presents the results of assessing the infectious process with the PEDIS system. Taking both groups into account it was found that grades 2 (40%) and 3 (40%) were the most common ones, followed by grade 4 (20%). Also, grade 3 was the most common one among patients that suffered an amputation (56% of the patients that suffered an amputation), while grade 2 was the most common one in the group of patients that only had surgical washing (64% of the patients that only had surgical washing). We note that according to the Spearman correlation coefficient, the relation between grade of infection (measured by the PEDIS scale) and grade of ulcer (measured by the Wagner system) was high, positive, and significant, r_s_ = 0.870, *p* < 0.001. Therefore, a higher grade of ulcer (according to the Wagner classification) corresponds to a higher grade of infection (according to the PEDIS scale).

Finally, Table 4 also shows the type of amputation suffered by the patients. Observe that 30 minor amputations were performed, while 15 patients suffered major amputations. In other words, 67% of all surgical treatments were minor amputations.

It is important to note that 11% of total patients developed an ulcer in the same anatomic site approximately 10 months after it had healed.

## Discussion

In our study we found that the great majority of our patients had a low level of education, since 76% of them were either illiterate or only had a complete or incomplete elementary education (grades 1–6). This has a great influence in the development of diabetic foot ulcers because the patients do not fully understand both the importance of taking care of the feet and the severity of having T2DM [20–22]. This is consistent with other studies [23–26]. In fact, recent studies have shown that people with a higher level of education are more aware of the severity of having T2DM, take better care of their condition, and participate more in programs devoted to the care of the diabetic foot and the prevention of foot ulcers [27,28]. Moreover, almost all of our patients had very low income and do not have health insurance. Also, we observed that most of the patients cannot follow a proper diet and adequate treatments for T2DM, glucose control, hypertension, kidney disease, and diabetic foot ulcers. In addition, we found that the group of patients that suffered an amputation (45%) had an average duration of T2DM of 13 years. This suggests that low levels of education and income combined with a long duration of T2DM contribute to the eventual development of infected, diabetic foot ulcers and the deterioration of the patient’s quality of life. These results are similar to those reported in other studies [26–32].

It is important to note that more than 50% of the patients were between 40 and 60 years old. Hence, the illness can diminish the patient’s life expectancy considerably. Moreover, as the patients’ age and their duration of T2DM increases, they are more susceptible to develop chronic ulcers and other complications such as neuropathy and macrovascular disease because the skin is more easily damaged. This is alarming because in our case, all of the patients presented with consistent characteristics such as anhidrosis and erythema. In addition, most of the patients are in a productive age and the disease can affect their work, leading to a burden to the family and public health institutions.

We found that men presented with a greater number of late comorbidities, such as social abandonment, poor nutrition, low socio-economic level, and abandonment of follow-up consultations. In addition, men also presented with the greater number of cases that did not follow the treatment indicated in the first consultation. These characteristics contribute to the development of more T2DM complications. To estimate the relative risk of amputation of a lower limb (RR) we evaluated the patients’ demographic variables (age, schooling, duration of T2DM, gender), history of smoking, and arterial hypertension (HAS). Males were more affected with an RR of 1.44 (IC 95%, 1.06–2.05, *p* = 0.043).

Our results indicate that one of the main characteristics of the diabetic foot ulcers in our patients was a high glucose level in blood, since we found that the level of HbA1c was high in 85% of the patients. High levels of glucose in blood lead to structural changes in the skin, joint capsules, and tendons. In particular, the skin no longer has the same stretching capacity and can break easily, leading to the formation of an ulcer [33–35]. Also, a high level of glucose combined with hypertension can lead to chronic kidney disease. Our results indicate that 43% of the patients had hypertension, while 47% of the patients had K/DOQI-II to IV. Of the latter, all patients with K/DOQI-III and IV suffered an amputation. Moreover, high levels of glucose in the blood are associated with a decrease in neutrophil activity and chemotaxis. As a result of this, the immune system’s response is reduced and the diabetic foot ulcers are more susceptible to infections [35–37]. In addition, we observed a low serum albumin (<2) in all of our patients. This entails a greater risk of amputation, since a low serum albumin implies poor nutrition and this can delay healing of the wound and, consequently, it can aggravate the lesion [38,39]. Again, the poor nutrition is due to the low socio-economic level of the great majority of the patients.

Another important result is that we found a high average leukocytosis in our patients, especially those that suffered an amputation. This is due to a response of the body to the infection and inflammation of the foot ulcer. Moreover, this infection and inflammation could be a result of a failure in the immune system’s response, a decrease in the peripheral circulation to the extremities, or a high level of glucose in blood. This has also been reported in other studies, where leukocytosis is considered a risk factor of amputation of lower extremities [40–43]. Also, infection and inflammation could play a role in the development of a peripheral arterial disease (PAD), aggravating the patient’s condition [44].

We found that the infection in most diabetic foot lesions was polymicrobial and that MRSA was the most common pathogen. This is alarming because many of the patients suffered severe infections from multi-resistant pathogens, a result that is similar to that of other studies [45,46].

We also found that 45% of the patients reported smoking. Although only 25 (56% of the 45 patients that reported smoking) suffered an amputation, a history of smoking is important in the development of diabetic foot ulcers because of the endothelial damage it produces. In particular, it has been reported that smokers suffer more from diabetic foot ulcers and have a higher risk of amputations than non-smokers [29,35,37,47,48].

The duration of the ulcers before hospitalization was 2.4–5 months and the area of the ulcers varied from 1.5 to 8 cm^2^. Most of the ulcers were found in the forefoot and most of the lesions were located on the plantar region of the foot. This probably occurs because a person usually exerts more pressure in that region of the foot while walking. Moreover, the most common triggering events for the foot lesions were the use of inadequate shoes, followed by the formation of blisters. The reason for the use of inadequate shoes is that most of the patients do not have enough money to buy special shoes and socks for diabetics.

The ulcer depth was assessed using Wagner’s classification. We found that the most frequent types of lesions were grades III (39%) and IV (34%). It is important to note that Table 4 indicates that some patients with grade IV or V lesions did not suffer an amputation. This was done through daily surgical washing that included debridement, antibiotics, and adequate care of the foot (see *Materials and methods*). Although these patients saved the lower extremity at the cost of losing a great part of the foot’s tissue, we found that avoiding the amputation produced a great psychological welfare in all them.

The degree of infection of the ulcers was classified using the PEDIS scale. We found that degrees 2 (40%) and 3 (40%) were the most common ones, a result similar to other studies [37]. After the study, we observed that 11 patients were readmitted to the hospital with more severe diabetic foot lesions, many of which eventually lead to amputations.

### Limitations of our study

It was difficult to assess the efficiency of the treatment of the foot lesions because many patients could not follow the treatment adequately due to economic reasons.

## Conclusions

We found there are delays in most of the patients treated at the hospital for both the diagnosis and the treatment of T2DM, as well as overall diabetic foot risk evaluation. This is a very alarming influence pertaining to our study because the majority of our patients have a very low level of education and income. The delays in diagnosis and treatment, combined with the low socio-economic level, produce a profound health and psychological detriment of patient well-being. Our results evidenced this because more than half of the patients arrive with advanced stages of diabetic foot ulcers. In fact, 45% of the patients suffered either a minor or major amputation. In addition, amputations further damage the socio-economic level and psychological welfare of the patients. The reason for this is that they may lose their job or they may not be able to perform their job adequately because, given the socio-economic level of the patients, it usually involves physical activity. Again, this leads to an economic and psychological burden for their families. Nevertheless, we also found that an efficient treatment can help in avoiding amputations, since 53% of grade IV and 25% of grade V lesions according to the Wagner system did not suffer an amputation. In particular, an effective antibiotic treatment and an education of the patient on the adequate care of their lesions are essential for this.

Due to the social and economic impact of diabetic foot complications in persons suffering from T2DM, ideally there should be an implementation of programs designed to provide adequate assistance and education to T2DM patients with a diabetic foot or at risk of developing it. This has the purpose of preventing complications, more advanced stages of deterioration, and amputation. These assistance and education programs should be directed to patients, family members, first contact physicians, and medical specialists treating diabetic patients. Given the current economic situation in Mexico, the only viable path appears to be that the physicians should provide an adequate education to the diabetic patient on the severity of his illness, the care of the feet, and the use of appropriate shoes and socks. This is essential in preventing the development of ulcers and eventual complications, especially in patients with a low level of education and income.
